# Supplementary material for: Circulating re-entrant waves promote maturation of hiPSC-derived cardiomyocytes in self-organized tissue ring
Source: Commun Biol. 2020 Mar 13;3:122. doi: 10.1038/s42003-020-0853-0 (PMC7070090; doi:10.1038/s42003-020-0853-0)
Supplement: Supplementary file 1 — Supplementary Information [file 42003_2020_853_MOESM1_ESM.pdf]

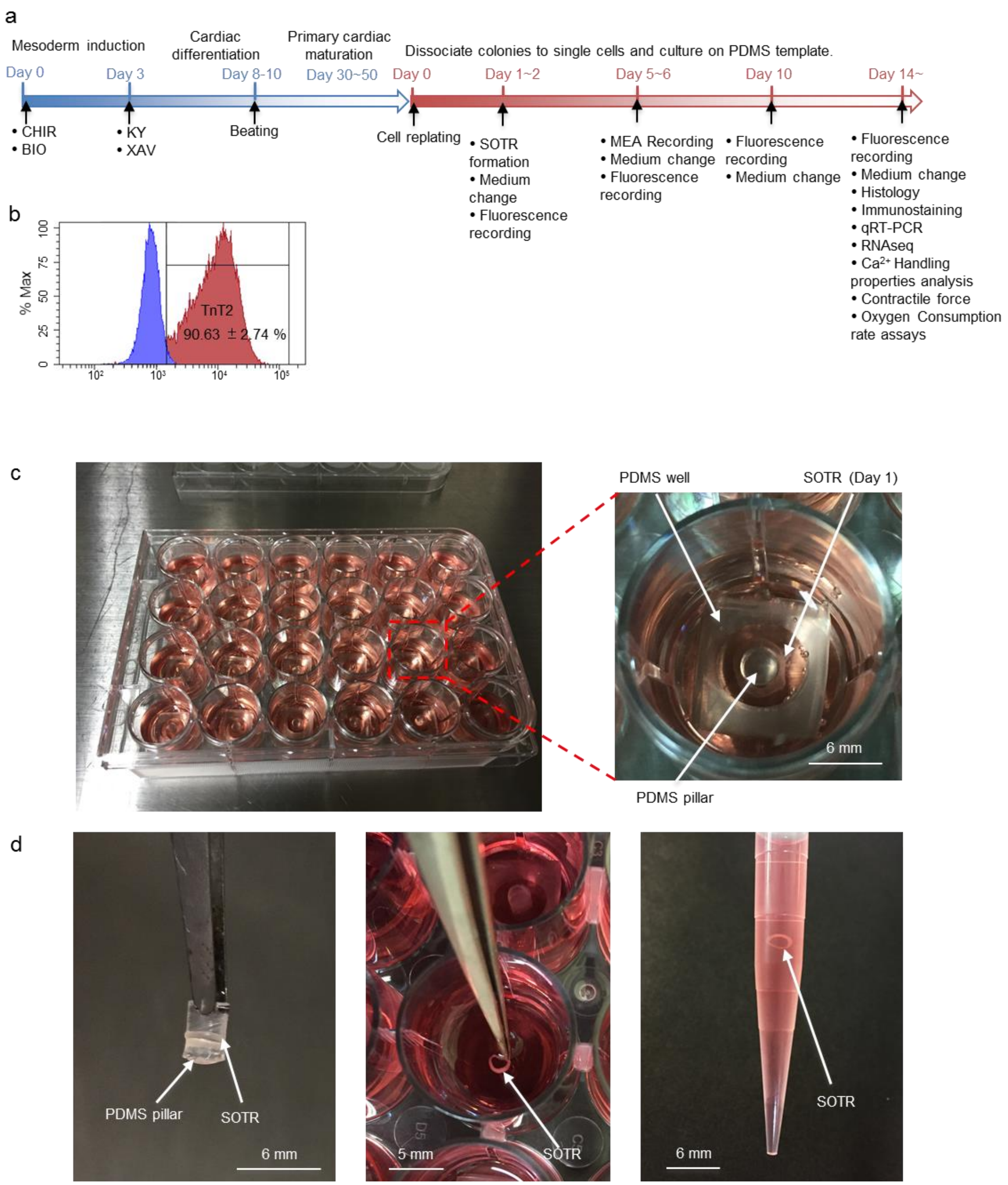

Supplementary Figure 1. SOTR formation. (a) Schematic representation of cardiomyocyte differentiation and functional assessment in SOTRs. (b) Flow cytometric analysis of the cardiomyocytes used for generating SOTRs. (mean ± s.d.; *n* = 4 biologically independent samples) (c) SOTRs in a 24-well culture dish. The

enlarged figure shows the PDMS well and pillar, between which the cells were plated and organized into a tissue ring. (d) SOTR with a PDMS pillar on day 14. The SOTR could be removed from the pillar and transferred with a pipette.

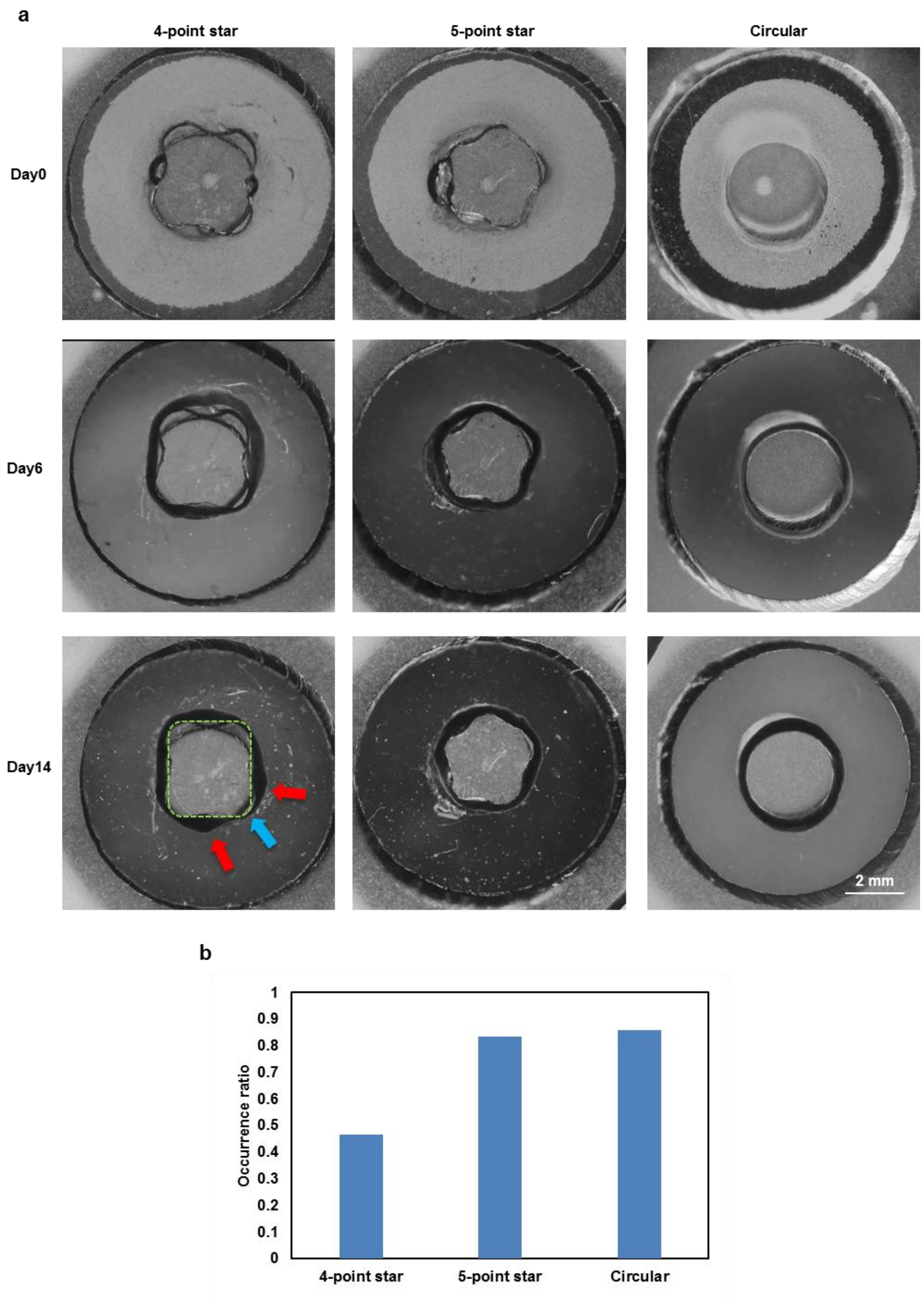

Supplementary Figure 2. hiPSC-derived cardiomyocytes in different templates. (a) The rings were formed within 6 days. The red and blue arrows indicate thick and thin

areas in tissue ring, respectively. (b) The percentage of occurrence for ReWs in different templates on day 6 (4-point star: n = 30; 5-point star: n = 6; Circular: n = 204 biologically independent samples)

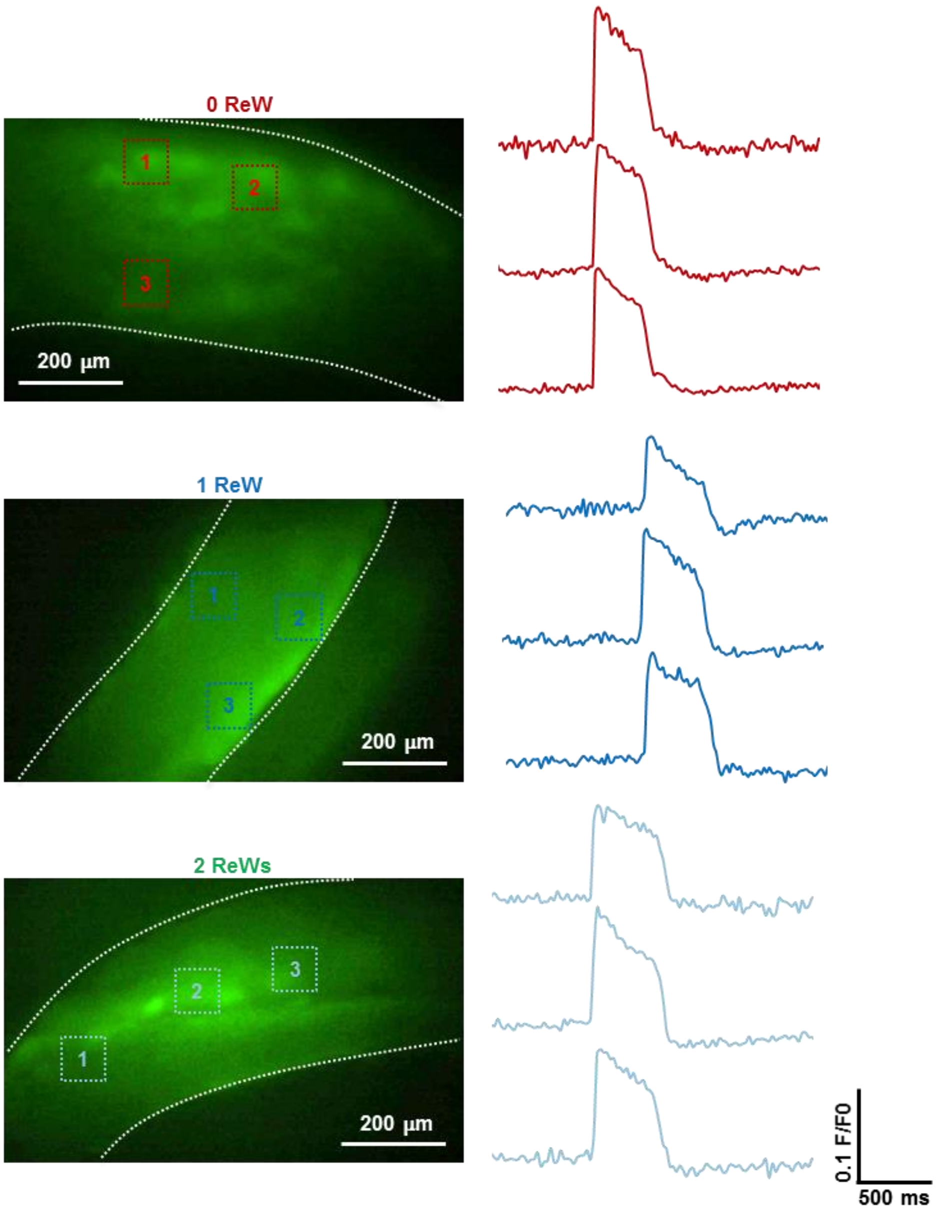

Supplemental Figure 3. Representative fluorescence images of SOTRs with zero, one, or two ReWs at day 6. cardiomyocytes were stained with FluoVolt voltage dye according to the manufacturer's manual. The dashed line marked the tissue area in the

images. The numbered squares mark the area where the membrane potential was recorded.

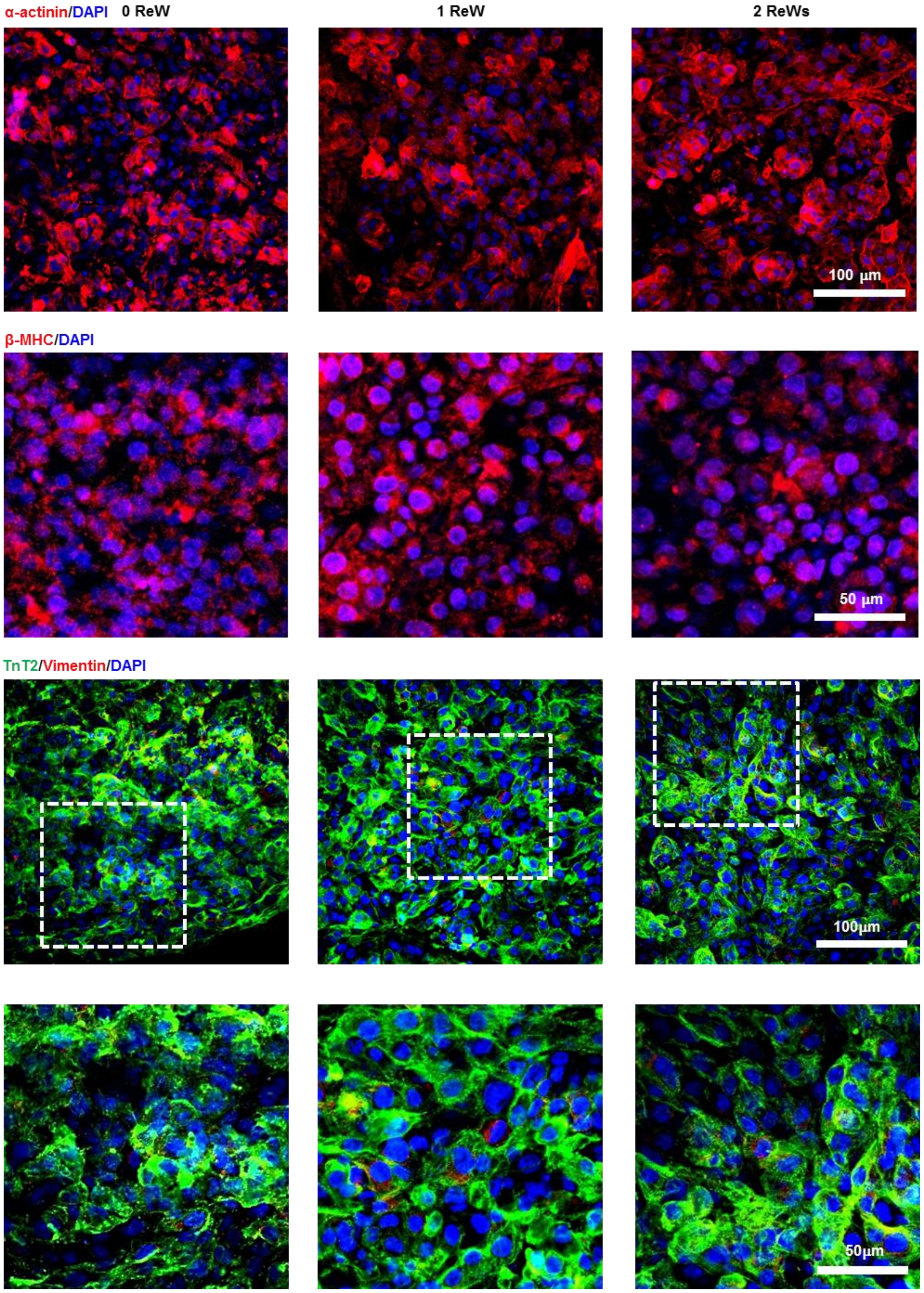

Supplementary Figure 4. Representative confocal images of SOTRs with zero, one, or two ReWs at day 2. cardiomyocytes were stained with anti-α-actinin (red), anti-β-MHC (red), anti-TnT2 (green), anti-Vimentin (red), and DAPI (blue).

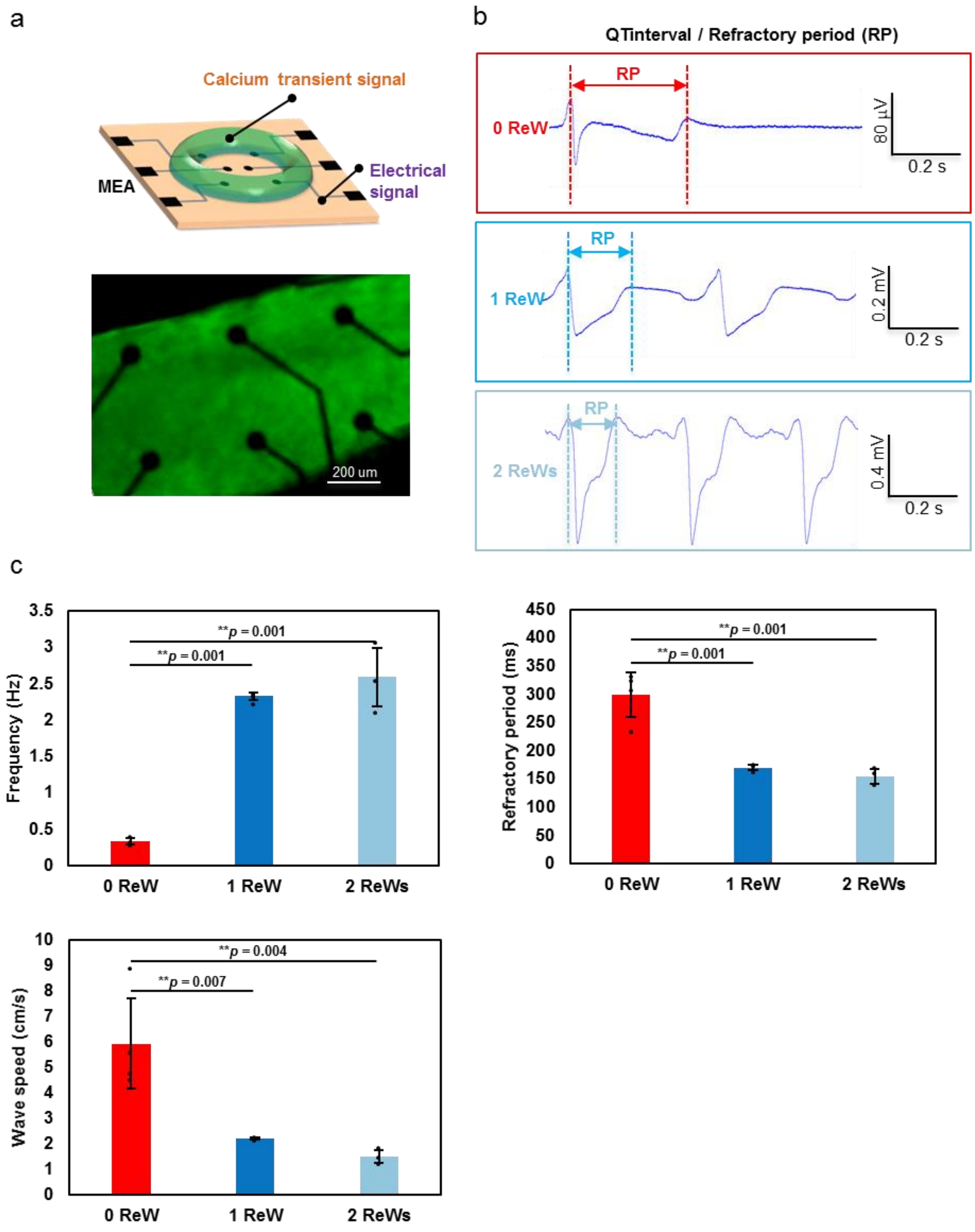

Supplementary Figure 5. Field potential recording by micro-electrode arrays. (a) Representative setup for Field potential **(**FP) recording by using electrodes underneath a SOTR on day 6. See also supplementary video 5. (b) Representative FP recorded by an electrode placed underneath a SOTR with or without ReWs. (c) Frequency, QT

interval (correlated with AP duration and refractory period), and wave speed in SOTRs with or without ReWs on day 6. [mean ± s.d.; 0 ReW (*n* = 4); 1 ReW (*n* = 4); 2 ReWs (*n* = 3 biologically independent samples)]. $^{**}P < 0.01$ (ANOVA).

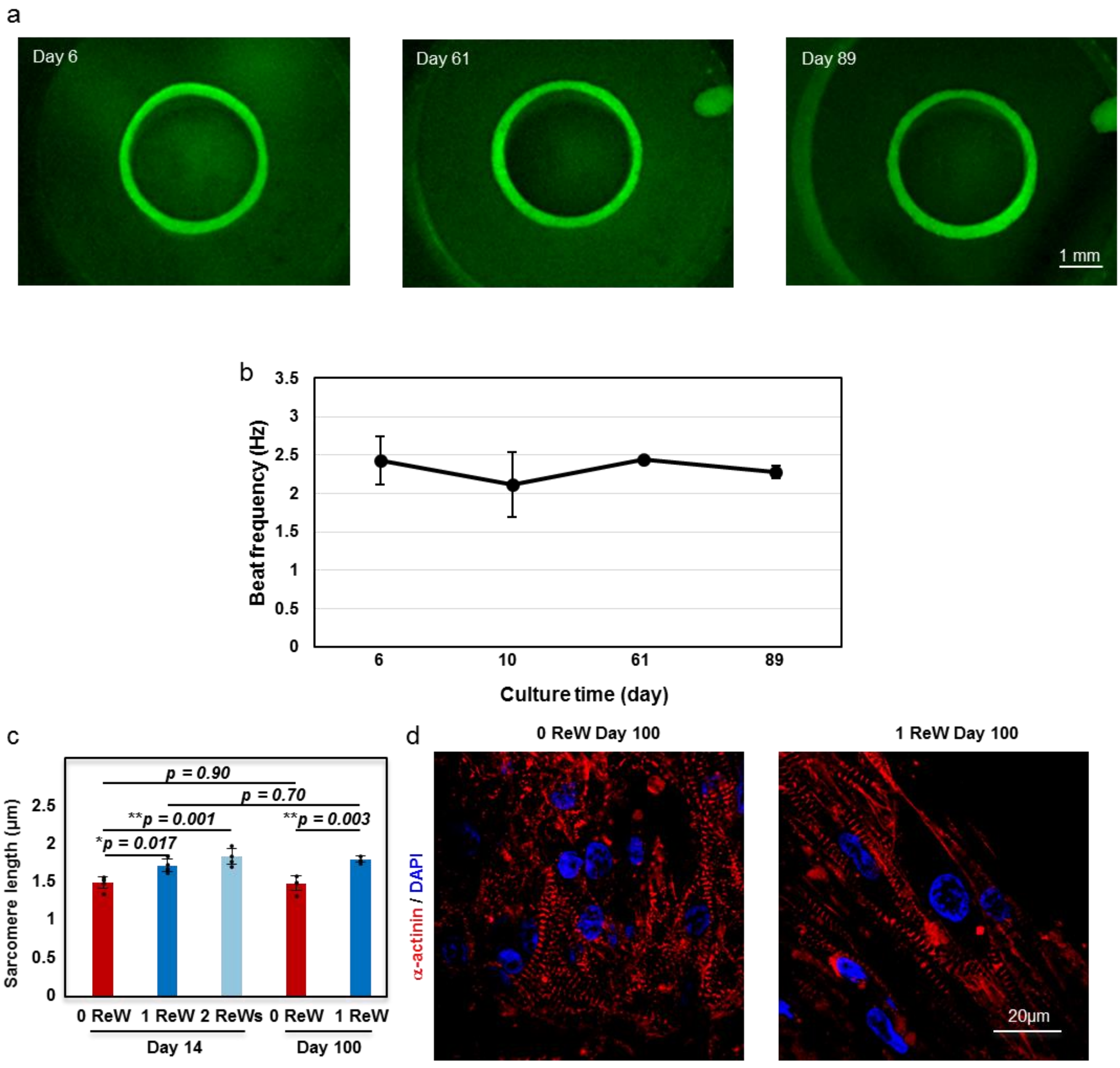

Supplementary Figure 6. Maintenance of ReWs in SOTRs for >89 days. (a) Fluorescence images of a SOTR with one ReW at different culture times. (b) The beat rate (Hz) of SOTRs with one ReW during long-term culture (mean ± s.d.; n = 3 biologically independent samples). (c) Sarcomere lengths of cardiomyocytes within SOTRs on day 14 and day 100 [mean ± s.d.; Day 14: 0 ReW (n = 5 biologically independent samples); 1 ReW (n = 5 biologically independent samples); 2 ReWs (n =

4 biologically independent samples); Day 100: 0 ReW (n = 4); 1 ReW (n = 3 biologically independent samples)]. *$P < 0.05$; **$P < 0.01$ (ANOVA).

a

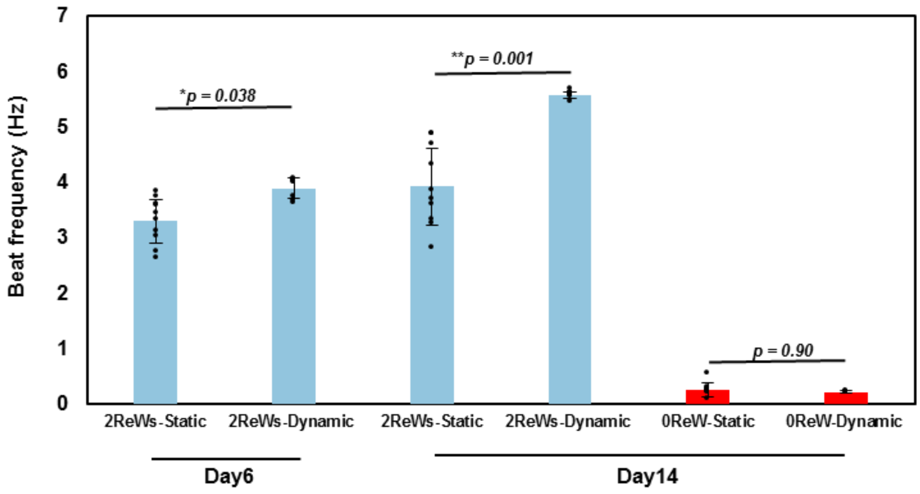

b

Day 6		Day 14	
Static	Dynamic	Static	Dynamic
85.79%	50%	71.51%	40%

Supplementary Figure 7. The comparison between dynamic and static culture of SOTRs. (a) Beat rates of SOTRs at different culture times, (mean ± s.d.; 2 ReWs-Static: n = 10; 2 ReWs-Dynamic: n = 8; 0 ReW-Static: n = 8; 0 ReW-Dynamic: n = 3 biologically independent samples.) **$P < 0.01$; ***$P < 0.001$ (ANOVA). (b) ReW occurrence ratio for static culture and dynamic culture (Static: n = 204; Dynamic: n = 20 biologically independent samples).

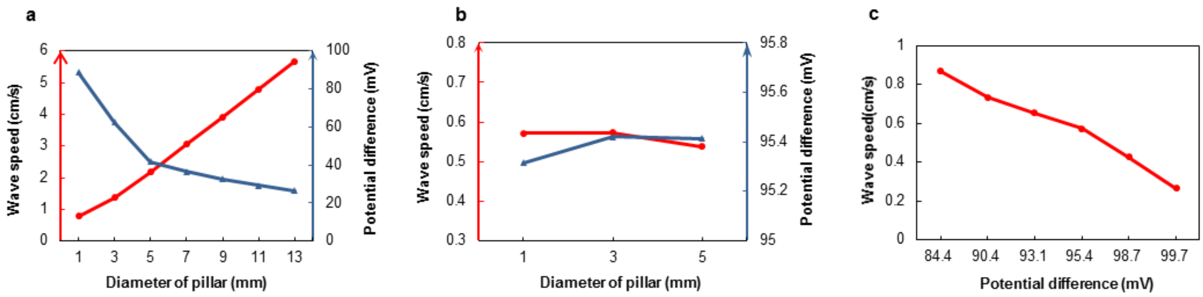

Supplementary Figure 8. Relationships between ReW speed and differences in membrane potential. (a) Relationships between ReW speed, differences in electrical potential between neighboring cells immediately before activation (at the front tip of the wave), and ring diameter. The blue line represents the ReW speed, and the orange line represents the difference in electrical potential. (b) The diameter of the ring does not affect the ReW velocity when cells lose the Funny current. The blue line represents ReW velocity, and the orange line represents the difference in electrical potential. (c) Differences in electrical potential affect the ReW speed.

a

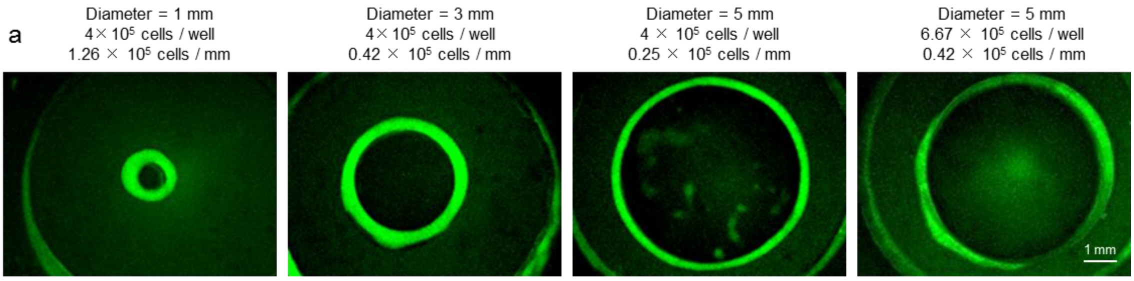

b

Cell density	Ratio of SOTRs with ReWs (Day 6)		
	Dia. = 1 mm	Dia. = 3 mm	Dia. = 5 mm
4×10^5 cells / well (fixed cell number)	13.51% (n=37)	91.02% (n=156)	48.15% (n=27)
0.42×10^5 cells / mm (fixed cell density)	N/A*		83.33% (n=60)

* Unable to form SOTRs

Supplementary Figure 9. SOTR formation using pillars with different diameters. (a) Fluorescence images of GCaMP3-positive SOTRs with different diameters at day 6. (b) Percentage of SOTRs with ReWs on day 6 after cell plating with different pillar diameters.

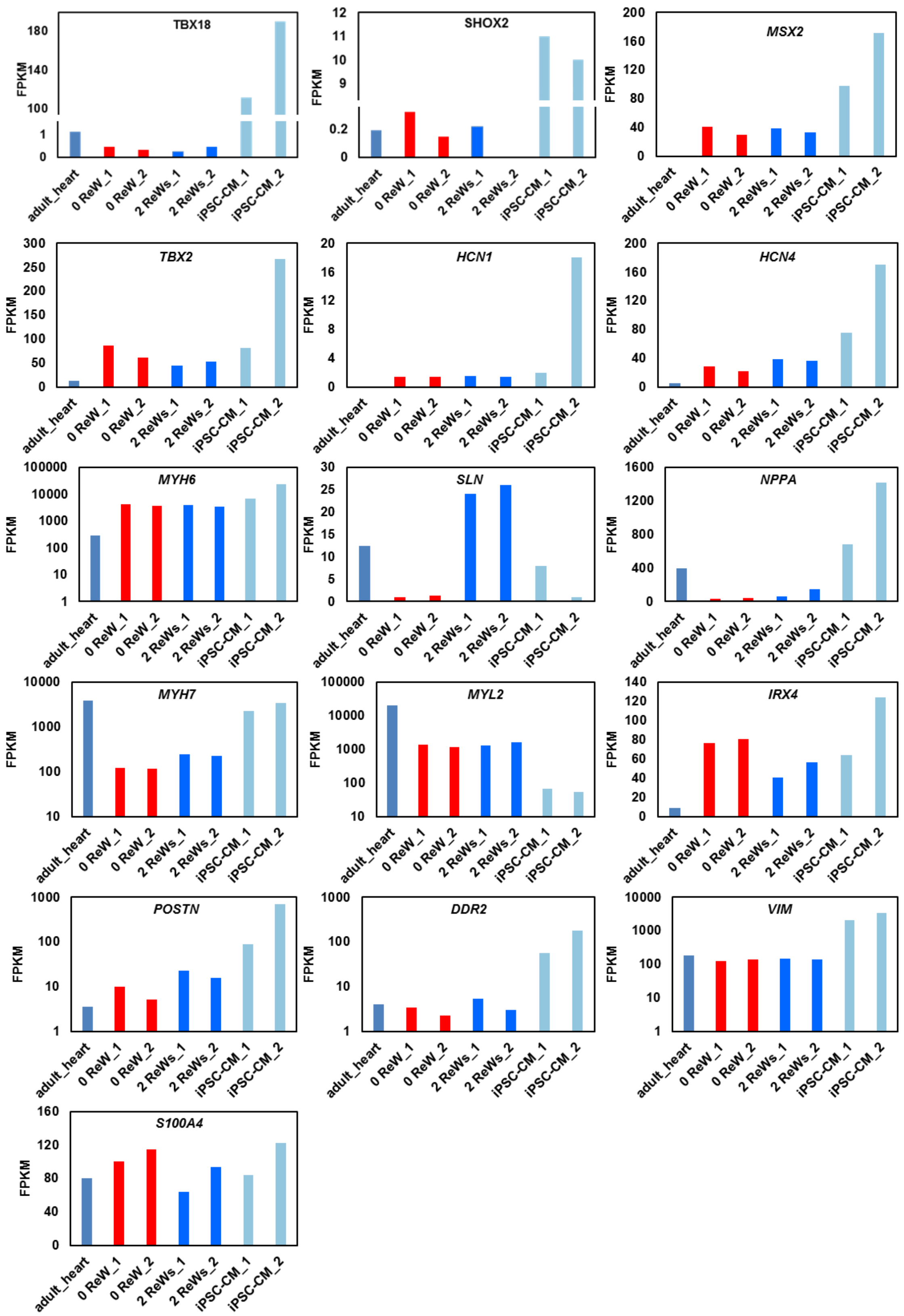

Supplementary Figure 10. FPKM values of cardiac markers from RNA-sequencing data. The samples include adult heart, two ReWs, zero ReW and other two

iPSC derived cardiomyocytes from a previous report[42]. The markers include nodal (*TBX18*, *SHOX2*, *MSX2*, *TBX2*, *HCN1*, and *HCN4*), atrial (*MYH6*, *SLN*, and *NPPA*), and ventricular (*MYH7*, *MYL2*, and *IRX4*) subtypes and fibroblast (*POSTN*, *DDR2*, and *VIM*, *S100A4*) related types.

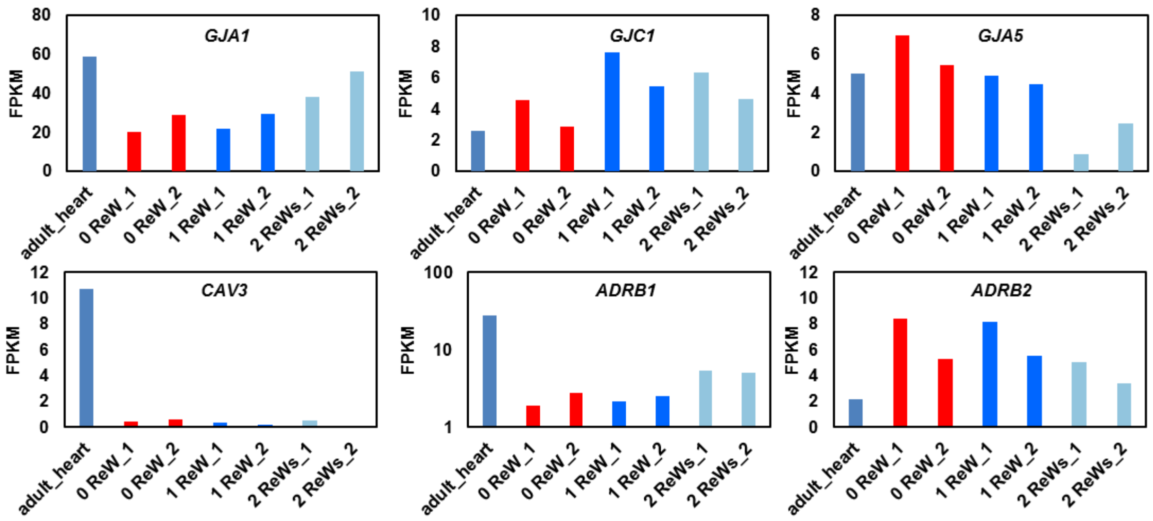

Supplementary Figure 11. FPKM values from RNA-sequencing data. The expression of *GJA1*, *GJC1*, *GJC1*, *CAV3*, *ADRB1* and *ADRB2* in different sample groups, which included adult heart, zero ReW, one ReW and two ReWs samples.

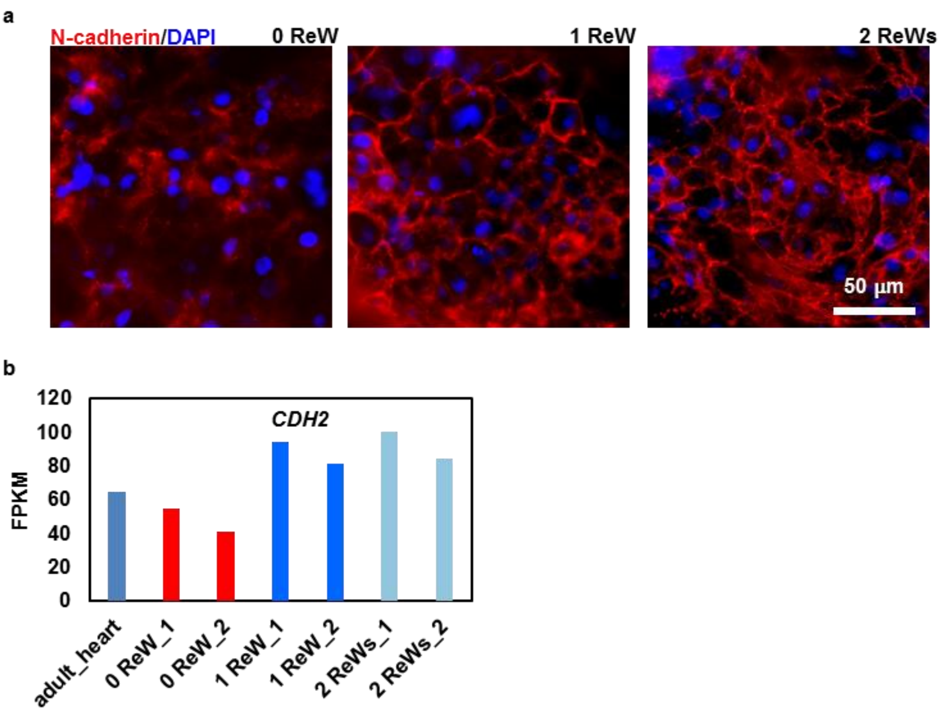

Supplementary Figure 12. N-cadherin expression in SOTRs with zero, one, or two ReWs at day 14. (a) cardiomyocytes were stained with anti-N-cadherin (red) and DAPI (blue). (b) The FPKM value of N-cadherin (CDH2) in different sample groups.

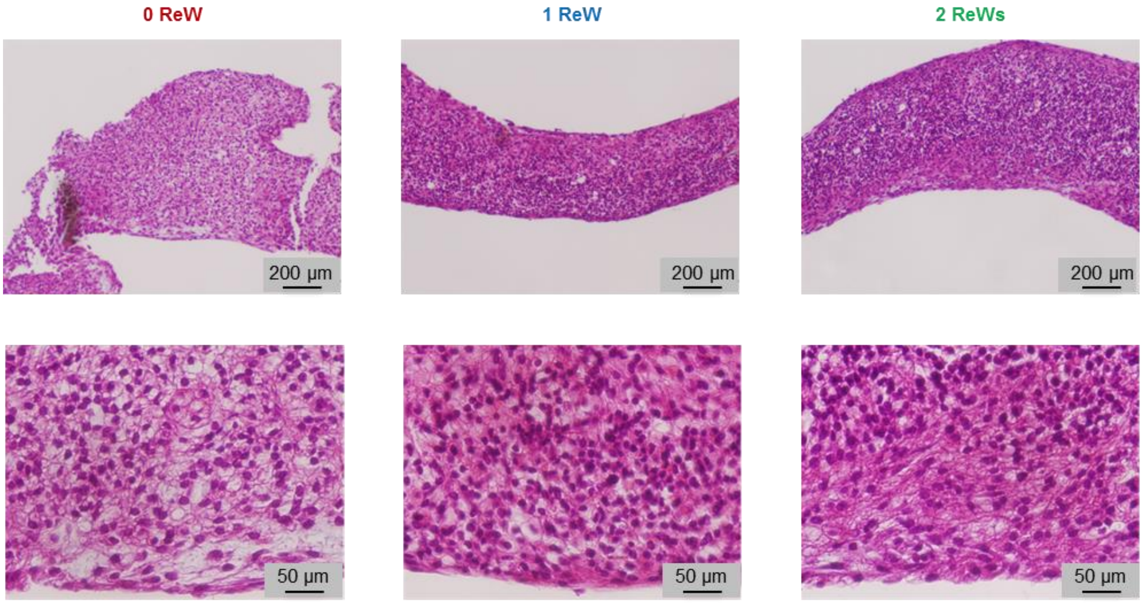

Supplementary Figure 13. Histology of SOTRs with different ReWs on day 6 after cell plating.

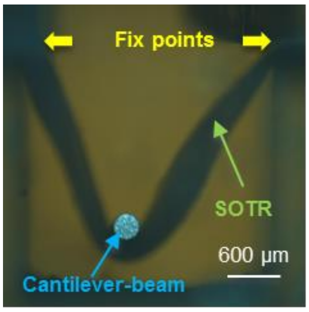

Supplementary Figure 14. The contractility force recording set up.

Supplementary Methods

Mathematical model

We constructed a mathematical model to simulate the phenomena observed in experiments. This model comprises two critical parts: 1) modeling of the action potential (AP) in a cell and 2) coupling between two neighboring cells. First, when we simulated the AP of a single human induced pluripotent stem cell-derived cardiomyocyte (hiPSC-CM), we referenced the classic Priebe & Beuckelmann (PB) model[1] of human ventricular AP based on the Hodgkin–Huxley formalism, with the equations derived from experimental data. To increase computational efficiency and stability, we referenced Panfilov's reformulated model[2] based on the PB model. Unlike human ventricular cells, hiPSC-CMs exhibit an ability to beat spontaneously, indicating that hiPSC-CMs are similar to sinoatrial node cells. Therefore, we added hyperpolarization-activated current[3], I_f, to reformulate the PB model. There was evidence that hiPSC-CMs differed significantly from adult ventricular CMs according to their reduced inward rectifier K^+ currents (I_{K1}) and the presence of prominent pacemaker currents (I_f) that contribute to hiPSC-CM automaticity[4]. Therefore, we changed the maximum conductance value, g_{K1}, of the inward rectifier K^+ currents (I_{K1}) in our mathematical model. A detailed description of the equations governing were described previously[1-3]. Second, there exists a conduction of electrical signals between neighboring cells. Previous studies treated this process using cable equations[5]:

$$\frac{\partial V}{\partial t} = \frac{1}{C_{\mathrm{m}}} \frac{a}{2 R_{\mathrm{i}} R_{\mathrm{CG}}} \frac{\partial^2 V}{\partial x^2} - \frac{1}{C_{\mathrm{m}}} I_{\mathrm{total}} \qquad (1)$$

$$R_{\mathrm{i}} = R_{\mathrm{myo}} + \frac{R_{\mathrm{g}}}{\Delta x}$$

where V is the membrane potential, C_m is the membrane capacity, I_{total} is the sum of individual membrane ionic currents in a single cell, a is the cell radius, Δx is the length of a single cell, R_{CG} is the ratio between capacitive and geometric areas, and R_i comprises the myoplasmic resistance, R_{myo}, and gap-junction resistance, R_g, as shown. By combining and modifying the above parts, we established a mathematical model of coupled hiPSC-CMs. To create the model in agreement with our experimental results, we set parameters as follows: $g_{Na} = 8$ nS pF^{-1}, $g_{to} = 0.35$ nS pF^{-1}, $g_K = 0.1$ nS pF^{-1}, $g_{K1} = 1.17$ nS pF^{-1}, $g_f = 0.08$ nS pF^{-1}, $R_g = 0.1\ \Omega$, and $R_{myo} = 0.007\ \Omega$ um^{-1}. We used N cells arranged next to each other in one dimension with periodic boundary conditions to simulate the self-organized tissue ring (SOTR) in the experiment.

The dependence of reentrant wave (ReW) speed on ring diameter

Both in the experiment and the simulation, ReW speed increased along with ring diameter (Fig. 3d). A key determinant of ReW speed was the difference in membrane potential between the yet-to-be-activated cell and the neighboring cell that activates it. All hiPSC-CMs are capable of spontaneously beating, and their APs change periodically. Therefore, maintaining spontaneous beating ability requires the Funny current (hyperpolarization-activated current[3]), I_f, which helps resting cells gradually elevate their APs beyond a threshold, followed by the initiation of depolarization. When stable ReWs appear in a ring, the time between two successive beats becomes shorter than that of its own spontaneous beat rate. This is because in the case of ReWs, the beating of a cell is due to activation by its neighbor, which would occur prior to its self-activation by the Funny current. The larger the ring diameter, the longer it will take the ReW to traverse it, and the longer a cell waits to be activated by its neighbor. This will bring the cell closer to its own self-activation point, resulting in a more rapid activation

when the neighbor is activated and a more rapidly propagating ReW. The above intuitive argument can be quantitatively expressed in the following equations.

$$T = n \times \Delta t \qquad (2)$$

$$\frac{\partial V}{\partial t} = -\frac{1}{C_m}\mathrm{HH}(V) + \frac{1}{C_m} D \frac{\partial^2 V}{\partial x^2}$$
$$\frac{\partial V_i}{\partial t} = -\frac{1}{C_m}\mathrm{HH}(V_i) + \frac{1}{C_m}\frac{D}{\Delta x^2}\{(V_{i+1} - V_i) - (V_i - V_{i-1})\}$$
$$\frac{\partial V_i}{\partial t} = -\frac{1}{C_m}\mathrm{HH}(V_i) + \frac{1}{C_m}\frac{D}{\Delta x^2}\{(V_i(t-\Delta t) - V_i(t)) - (V_i(t) - V_i(t+\Delta t))\}$$
$$\frac{\partial V_i}{\partial t} = -\frac{1}{C_m}\mathrm{HH}(V_i) + \frac{1}{C_m}\frac{D}{\Delta x^2}\{(V_i(t-\frac{T}{n}) - V_i(t)) - (V_i(t) - V_i(t+\frac{T}{n}))\} \qquad (3)$$

$$V_i(t) = V_i(t+T) \qquad (4)$$

T: the time for one ReW to travel around the ring

n: the number of cells

Δt: the difference in time between the point at which the electrical signal arrives at two adjacent cells

V: membrane potential

V_i: the membrane potential of the ith cell

HH(*V*): derivation of V and I using the HH equation

D: diffusion coefficient

Given the diameter of the ring, the number of cells is determined (*n*). Using the periodic boundary condition (Eq. 4), the above equations can be solved to find a

relationship between the cycle time of the membrane potential of a single cell and the diameter of the ring (Supplementary Figure 8a).

To verify the accuracy of the above explanation, we performed two simulations. First, the Funny current resulted in different AP differences between the two adjacent cells at the wave front in rings with different diameters. Therefore, we deleted the Funny current in every cell, resulting in the loss of spontaneous beating ability and an inability of its AP to change in the resting state over time. This meant that whenever a cell was activated, the difference in AP between the activating cell and the activated cell would be the same, regardless of the diameter of the ring. In this simulation, we found that the diameter of the ring did not affect ReW velocity, suggesting that transmitted electrical signals in different ring diameters had no effect on speed due to their shared difference in electrical potential (Supplementary Figure 8b).

Second, we artificially raised the minimum membrane potential of cells in the resting state and maintained their excitable characteristics (with no Funny current). Unlike the first simulation, we established gradients of differences in electrical potential between activating and activated cells. After measuring the propagation velocity of the ReWs, we found that the propagation speed decreased as the difference in electrical potential increased (Supplementary Figure 8c).

These two simulations demonstrated that different ReW speeds in rings of different diameters were caused by differences in the electrical potential between activated and activating cells. The smaller the ring diameter, the greater the difference in electrical potential and the smaller the propagation speed.

Reference

1. Priebe, L. & Beuckelmann, D. J. Simulation study of cellular electric properties in heart failure. *Circ Res* **82,** 1206–1223 (1998).
2. Panfilov, a V. a. of Human Ventricular Cells. **281,** 2296–2308 (2002).
3. Verkerk, A. O., Van Borren, M. M. G. J. & Wilders, R. Calcium transient and sodium-calcium exchange current in human versus rabbit sinoatrial node pacemaker cells. *Sci. World J.* **2013,** (2013).
4. Karakikes, I., Ameen, M., Termglinchan, V. & Wu, J. C. Human Induced Pluripotent Stem Cell-Derived Cardiomyocytes: Insights into Molecular, Cellular, and Functional Phenotypes. *Circ. Res.* **117,** 80–88 (2015).
5. Shaw, R. M. & Rudy, Y. Ionic mechanisms of propagation in cardiac tissue. Roles of the sodium and L-type calcium currents during reduced excitability and decreased gap junction coupling. *Circ. Res.* **81,** 727–741 (1997).
