# Supplementary material for: Circulating re-entrant waves promote maturation of hiPSC-derived cardiomyocytes in self-organized tissue ring
Source: Commun Biol. 2020 Mar 13;3:122. doi: 10.1038/s42003-020-0853-0 (PMC7070090; doi:10.1038/s42003-020-0853-0)
Supplement: Supplementary file 9 — Description of Additional Supplementary Files [file 42003_2020_853_MOESM9_ESM.pdf]

Description of Additional Supplementary Files

File Name: Supplementary Movie 1

Description: Reentrant waves in the self-organized tissue ring: This video shows the phase contrast and fluorescence images (calcium transient) of a self-organized tissue ring with 2 reentrant waves.

File Name: Supplementary Movie 2

Description: Self-organized tissue rings with different numbers of reentrant waves: This video shows the fluorescence images (calcium transient) of self-organized tissue rings with 0, 1 and 2 reentrant waves, respectively.

File Name: Supplementary Movie 3

Description: Self-organized tissue ring on microelectrode array: This video shows the self-organized tissue ring with 1 reentrant wave integrated on a microelectrode array for simultaneous recording of electrical and optical signals.

File Name: Supplementary Movie 4

Description: Mathematical model simulation of the self-organized tissue ring**:** This video shows the membrane potential of the SOTR simulation for 4 cases: 0 reentrant wave, 1 reentrant wave, 2 reentrant waves and 3 reentrant waves. In each case, both the initial random state and the emerging stable reentrant wave state are shown.

File Name: Supplementary Movie 5

Description: The emergence of stable reentrant waves in self-organized tissue rings: This video shows the fluorescence images (calcium transient) of three SOTR samples. In each sample, both the initial state and the emerging stable reentrant wave state are shown.

File Name: Supplementary Movie 6

Description: Electrical stimulation on 0 reentrant wave ring: This video shows the 0 reentrant wave ring paced by electrical stimulation at day 14 and 1 reentrant wave is induced within the tissue.

File Name: Supplementary Data

Description: This Excel workbook contains source data used to plot graphs as follows:

Figure 1c; Figure 1f; Figure 1g; Figure 1h; Figure 2b; Figure 2c; Figure 2d; Figure 2e; Figure 2f; Figure 3f; Figure 3g; Figure 4d; Figure 5a; Figure 5b; Figure 5d; Figure 5f; Supplementary Fig1b; Supplementary Fig2b; Supplementary Fig5c; Supplementary Fig6b; Supplementary Fig6c; Supplementary Fig7a; Supplementary Fig8a; Supplementary Fig8b; Supplementary Fig8c; Supplementary Fig10-12;
