# Supplementary material for: Circulating re-entrant waves promote maturation of hiPSC-derived cardiomyocytes in self-organized tissue ring
Source: Commun Biol. 2020 Mar 13;3:122. doi: 10.1038/s42003-020-0853-0 (PMC7070090; doi:10.1038/s42003-020-0853-0)
Supplement: Supplementary file 10 — Reporting summary [file 42003_2020_853_MOESM10_ESM.pdf]

nature research

Corresponding author(s): Li Liu
Last updated by author(s): Feb 6, 2020

Reporting Summary

Nature Research wishes to improve the reproducibility of the work that we publish. This form provides structure for consistency and transparency in reporting. For further information on Nature Research policies, see Authors & Referees and the Editorial Policy Checklist .

Statistics

For all statistical analyses, confirm that the following items are present in the figure legend, table legend, main text, or Methods section.

n/a	Confirmed	
☐	☒	The exact sample size (*n*) for each experimental group/condition, given as a discrete number and unit of measurement
☐	☒	A statement on whether measurements were taken from distinct samples or whether the same sample was measured repeatedly
☐	☒	The statistical test(s) used AND whether they are one- or two-sided<br>*Only common tests should be described solely by name; describe more complex techniques in the Methods section.*
☒	☐	A description of all covariates tested
☒	☐	A description of any assumptions or corrections, such as tests of normality and adjustment for multiple comparisons
☐	☒	A full description of the statistical parameters including central tendency (e.g. means) or other basic estimates (e.g. regression coefficient) AND variation (e.g. standard deviation) or associated estimates of uncertainty (e.g. confidence intervals)
☐	☒	For null hypothesis testing, the test statistic (e.g. *F*, *t*, *r*) with confidence intervals, effect sizes, degrees of freedom and *P* value noted<br>*Give P values as exact values whenever suitable.*
☒	☐	For Bayesian analysis, information on the choice of priors and Markov chain Monte Carlo settings
☐	☒	For hierarchical and complex designs, identification of the appropriate level for tests and full reporting of outcomes
☒	☐	Estimates of effect sizes (e.g. Cohen's *d*, Pearson's *r*), indicating how they were calculated

Our web collection on statistics for biologists contains articles on many of the points above.

Software and code

Policy information about availability of computer code

Data collection	ImageJ, Matlab
Data analysis	In this work, we have used two softwares (ImageJ, Matlab) previously described in published literatures, which are both referred in the manuscript.

For manuscripts utilizing custom algorithms or software that are central to the research but not yet described in published literature, software must be made available to editors/reviewers. We strongly encourage code deposition in a community repository (e.g. GitHub). See the Nature Research guidelines for submitting code & software for further information.

Data

Policy information about availability of data

All manuscripts must include a data availability statement. This statement should provide the following information, where applicable:
- Accession codes, unique identifiers, or web links for publicly available datasets
- A list of figures that have associated raw data
- A description of any restrictions on data availability

All data generated or analysed during this study are included in this article (and its supplementary information files)

Field-specific reporting

Please select the one below that is the best fit for your research. If you are not sure, read the appropriate sections before making your selection.

☒ Life sciences ☐ Behavioural & social sciences ☐ Ecological, evolutionary & environmental sciences

Life sciences study design

All studies must disclose on these points even when the disclosure is negative.

Sample size	The sample size was determined in reference to a number of previous works in the same field. No sample size calculations were performed. For all experiments, at least three biological replicates or analyses were carried out.
Data exclusions	No data were excluded from the analyses.
Replication	All attempts at replication were successful.
Randomization	This is not relevant to our study because we are investigating the same tissue with different stimulation methods. No randomization is needed.
Blinding	this is not relevant to our study because we are investigating the groups with certain experimental conditions. No blinding is needed.

Reporting for specific materials, systems and methods

We require information from authors about some types of materials, experimental systems and methods used in many studies. Here, indicate whether each material, system or method listed is relevant to your study. If you are not sure if a list item applies to your research, read the appropriate section before selecting a response.

Materials & experimental systems

n/a	Involved in the study	
☐	☒	Antibodies
☐	☒	Eukaryotic cell lines
☒	☐	Palaeontology
☒	☐	Animals and other organisms
☒	☐	Human research participants
☒	☐	Clinical data

Methods

n/a	Involved in the study	
☒	☐	ChIP-seq
☐	☒	Flow cytometry
☒	☐	MRI-based neuroimaging

Antibodies

Antibodies used	Anti-TnT2 (mouse monoclonal IgG, 1:200; Santa Cruz Biotechology, SC-20025), anti-alpha-actinin (mouse monoclonal IgG, 1:1000; Sigma: A7811) or anti-beta-MHC (mouse MYH7 monoclonal IgM, 1:100; Santa Cruz Biotechnology, SC-53089).
Validation	All antibodies were used in this study according to the profile of manufacturer.

Eukaryotic cell lines

Policy information about cell lines

Cell line source(s)	Human iPS cell line (GCaMP-253G1) was created by Dr. Yuji Shiba (Shinshu University)
Authentication	None of the cells used have been authenticated.
Mycoplasma contamination	Cell lines were checked. Cell were negative for mycoplasma contamination.
Commonly misidentified lines (See ICLAC register)	No commonly misidentified cell lines were used.

Flow Cytometry

Plots

Confirm that:

☒ The axis labels state the marker and fluorochrome used (e.g. CD4-FITC).

☒ The axis scales are clearly visible. Include numbers along axes only for bottom left plot of group (a 'group' is an analysis of identical markers).

☒ All plots are contour plots with outliers or pseudocolor plots.

☒ A numerical value for number of cells or percentage (with statistics) is provided.

Methodology

Sample preparation	Sample preparation listed in Mthods
Instrument	FACS Canto II flow cytometer (BD Biosciences, 409 USA)
Software	FlowJo softwere (Treestar Inc., USA)
Cell population abundance	Cell sorting not employed
Gating strategy	Please see figure.

☒ Tick this box to confirm that a figure exemplifying the gating strategy is provided in the Supplementary Information.
